# S100, bcl2 and myeloperoxid protein expirations during periodontal inflammation

**DOI:** 10.1186/s12903-015-0077-8

**Published:** 2015-08-07

**Authors:** Yevhen Kuzenko, Anatoliy Romanyuk, Antonina Politun, Ludmila Karpenko

**Affiliations:** Department of Pathological Anatomy, Sumy State University, Sumy, Ukraine; Head of the Department of Therapeutic Dentistry, Kiev University UANM, Kyiv, Ukraine

## Abstract

**Background:**

Periodontal inflammation is characterized by injuries in collagen, epithelial, bone tissues. The hypotheses to be tested were relationship between the s100, bcl2 and myeloperoxidase in gingival tissues (MPO does affect the level of s100, bcl2). The object of this study was to investigate of s100 expression, bcl2 expression and myeloperoxidase expression in periodontal inflammation.

**Methods:**

27 patients (giant-cell epulis) and 30 patients (acute and chronic inflammations) were included in the study for s100 expression, bcl2 expression and myeloperoxidase expression by immunohistochemistry and hematoxylin - eosin.

**Results:**

Giant-cells in epulis positivity for myeloperoxidase has been observed in 100 % However, only 75.31 % of giant-cells were positive for bcl2 expression. Acute 98.2 %, and chronic 89.28 % inflammation was a significant positive for myeloperoxidase. The immunohistochemical findings of s100, bcl 2 and myeloperoxidase in epithelial layers have showed the result of 100 %, 82,2 %, 100 % positive cells in acute and 100 %, 78.25 %, 100 % in chronic process of inflammation respectively.

**Conclusion:**

The results indicate that the pathogenesis of periodontal inflammation might involve inhibition of cell death, through the overexpression of bcl-2, due to identifying factors myeloperoxidase (result in the DNA damage by the product of catalysis). The highest levels of s100 activity have been found at sites with chronic inflammation.

## Background

Bacterial infections are the most important etiologic agents involved in acute and chronic periodontitis, it is multifactorial disease that leads to the destruction of the bone periodontium [1]. Inflammatory cells infiltration has been resulted from periodontal plasma cells: neutrophil, T- & B- lymphocytes and macrophages [2]. Periodontal lesions have been characterized by a persistence of infiltrating inflammatory cells, which may be responsible for the bone collagens resorcinol. Research by Baelum V. & Lopez R. [3] demonstrated that periodontal disease affects between 10 % and 15 % of the world’s population, being the greatest cause of tooth loss.

Inflammatory cells (plasma cells) have expressed in myeloperoxidase. Polymorphonuclear neutrophils are largely expressed the myeloperoxidase in plasma cells [4]. The myeloperoxidase gene is located on chromosome 17 (17q23.1) [5].

Myeloperoxidase (MPO) have catalyzed the synthesis of microbicidal hypochlorous acid enabling the defence against bacteria [6]. Furthermore, plasma cells synthesize hypochlorous acid from H_2_O_2_ and NaCl. Hydroxyl radical (−OH) is mostly active in damaging important molecules such as DNA proteins and lipids [7]. Hydrogen peroxide (H_2_O_2_) being a potent agent of oxygen species, is capable of crossing the nuclear membrane and damaging the DNA. [8] There is growing support for the claim that inflammation induces DNA damage which leads to apoptosis in periodontal cells [9] Furthermore, the apoptotic stimuli can trigger apoptosis via different mechanisms, including specific cell death receptors and ligands, such as cd95 [10], stress signals, inducing molecules directly or indirectly in apoptosis, via p53. Bcl2 is a member of anti-apoptotic family proteins that can prevent or reduce cell death induced by a variety of stimuli [11]. The BCL-2 gene was identified in the chromosome human t(14;18) [12]. The intrinsic death pathway is initiated by the mitochondrial release of cytochrome *c*, a process that is inhibited by anti-apoptotic bcl2 proteins [13].

S100 proteins have expressed in the neutrophils cytosol, monocytes, activated macrophages, and keratinocytes and released during activation or death of these cells. The S100 gene family includes at least 13 members that locate as a cluster on chromosome 1q21 [14]. s100 proteins also known as L1 antigens, calgranulin A and B, macrophage migration inhibitory, factor-related protein (MRP) and cystic fibrosis antigen, have several functions in inflammatory reactions [15].

The results by Sun-Hee Heo et. al. [16] have shown the expression patterns of S100A2 in gingival tissues during bacterial lipopolysaccharide stimulation. S100A2 expression was upregulated by bacterial lipopolysaccharide.

We have hypothesis states that there is the relationship between the s100, bcl2 and myeloperoxidase in gingival tissues (MPO does affect the level of s100, bcl2).

The aim of this study is to compare the expression levels of s100, bcl2 and MPO in gingival tissues on different stages of periodontal disease and compared to the giant-cell epulis.

## Methods

### Patient selection and of gingival tissues collection

The study samples have included the periodontal and epulis tissues of the patients. The subjects were divided into two equal groups:

Patient Group (Group 1). Giant cell granuloma specimens were collected from 27 people who had a morphologically diagnosis of giant cell granuloma (eight males and 14 females, age range 30 to 70 years, mean age, 47,51 ± 12.37 years).

Control group (Group 2) consisted of 30 patients who had died in Sumy Regional Hospital. The patients had various somatic diagnoses (not atherosclerotic complications) and dental – parodontit. 17 males and 13 females, age range 43 to 69 years, mean age, 57.33 ± 8.31 years have been investigating. The specimens of overgrown gingiva were collected during jaws sawing procedures. After group 2 tissue samples stained by hematoxylin eosin foo, all samples were divided in two groups (acute and chronic). Control group has two subgroups. Acute subgroups - 13 (5 males and 3 females, age range 43 to 69 years, mean age, 54.38 ± 7.9 years). Chronic subgroups - 17 (7 males and 15 females, age range 44 to 68 years, mean age, 59.58 ± 8.11 years).

Informed written consent was obtained from all study subjects in accordance with guidelines established by the Ukraine Health Council. The present study was approved by the Sumy State University (Protocol no. 5/2012).

### Hematoxylin and eosin (H&E)

Stains have been used for at least a century and are still essential for identifying various tissue types and the morphologic change.

### Immunostainings

For s100, bcl2 and MPO have been performed formalin-fixed (pH 7,4) tissue. Paraffin-embedded tissue sections have been treated dy mouse monoclonal anti-s100, anti-bcl2 and anti-myeloperoxidase (Thermo Fisher Scientific UK). Briefly, 4 μm thick tissue sections were dewaxed in xylene and were placed in to water through graded alcohols. Antigen retrieval has been performed by microwaving slides in 10 mM citrate buffer (pH 6.2) for 30 min at high power, according to the manufacturer’s instructions. To remove the endogenous peroxidase activity, the sections have been treated with freshly prepared 1.0 % hydrogen peroxide in the dark for 30 min at 37 °C temperature. Non-specific antibody binding was blocked by means of blocking serum. The sections were incubated for 30 min, at 37 °C temperature, with the primary antibodies against s100, bcl2 and myeloperoxidase diluted 1:100 in phosphate buffered saline (PBS) pH 7.2 then a triple washing with PBS follows. Anti-(mouse IgG)–horseradish peroxidase conjugate (1:40 000 dilution) has been fulfilled for the detection of the S100, Bcl2 and MPO primariy antibodies, then the sections were incubated for 20 min, at 37 °C temperature. The colour was visualized by DAB.

The appearance of the positive factors was detected semiquantitatively by counting of positive giant cells in visual field.

The data were analysed using STATISTICA 8.0 software, user version STA862D175437Q. The results have been presented as mean ± SD. The normalize test have been use before analysis of the data. Also, the non-parametric Student method was applied to perform a simple comparative analysis. The value of P < 0.05 have been considered to be a significant.

## Results

The groups 1 and 2 of men and women consisted mostly age range 30- to 70-years. Group 1 giant cells occurred in the lower jaw (55 %) more frequently than in the upper jaw. In the group 2 the patients have been divided into 13 with acute and 17 with chronic inflammations.

**Fig 1:**
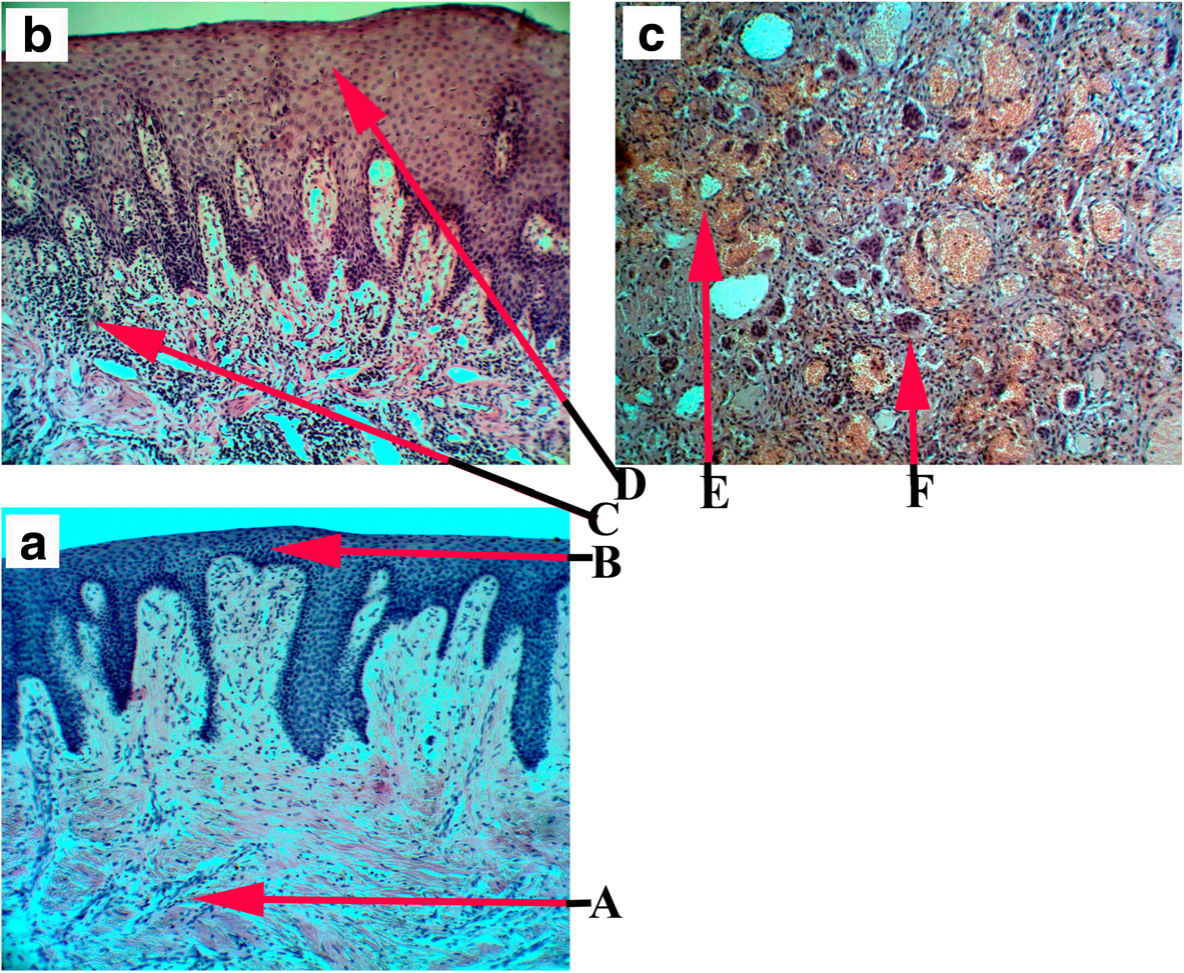
The periodontal tissues Haematoxylin and Eosin stained (x100 magnification) A (**a**) – pint-size cells infiltration with superimposed edema, B (**a**) – layers of the epithelium, C (**b**) - great cells infiltration with superimposed edema, D (**a**) – epithelial proliferation, E (**c**) - hemorrhage zone, F (**c**) - Giant cells

In Fig. 1a A we observed low-size cell infiltration in acute inflammation. Significant cell infiltration and proliferating epithelium (Fig. 1b) appeared to be more intensive in the chronic inflammation. Acute and chronic inflammatory cells are circulating leukocytes, plasma cells and tissue macrophages.

Peripheral giant cell epulis is shown in Fig. 1c Microscopic examination has revealed the tissue with the abundance of giant-cells (Fig. 1c F), fibrous connective tissue, areas of haemorrhages (Fig. 1c E) and few capillaries. There was no sign of malignancy. Chronic inflammation when the macrophages fail to disintegrated various particles, fuse together and form multinucleated giant cells. Besides, morphologically distinct giant cells also appear in some tumours also.

**Fig 2:**
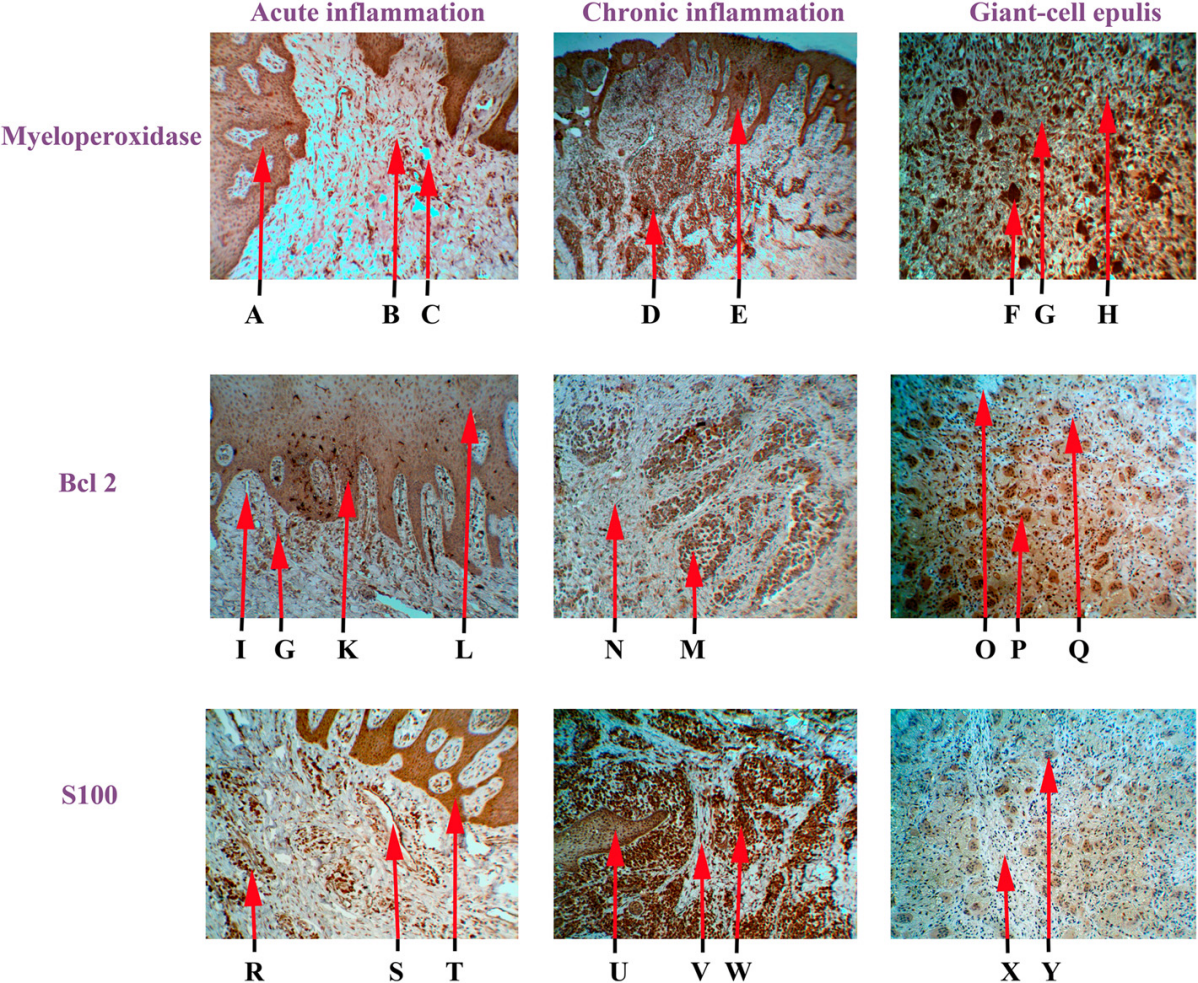
Expression of s100, bcl2and myeloperoxidase in gingival tissue (x100 magnification): A – Layers of the epithelium with myeloperoxidase expression, B – Blood cells infiltration with myeloperoxidase expression, C – Pericellular and perivascular edema, D – Blood cells infiltration with myeloperoxidase expression, E – Layers of the epithelium with myeloperoxidase expression and proliferation, F – Giant cells with myeloperoxidase expression, G – Fibroblastic stroma, H – Blood cells infiltration with myeloperoxidase expression, I – Pericellular and perivascular edema, G – Blood cells infiltration with Bcl 2expression, K – Layers of the epithelium with high level of bcl 2 expression, L – Layers of the epithelium with bcl 2expression, N – Fibroblastic stroma, M – Blood cells infiltration with bcl 2 expression, O – Pericellular and perivascular edema, P – Giant cells with bcl 2 expression, Q – Blood cells infiltration with low bcl 2expression, R – Blood cells infiltration with s100 expression, S – Pericellular and perivascular edema, T – Layers of the epithelium with s100 expression, U – Layers of the epithelium with low s100 expression, V – Fibroblastic stroma, W – Blood cells infiltration with s100 expression, X – Fibroblastic stroma, Y – Giant cells with “poor” s100 expression

S100, Bcl2 and MPO expressed in giant-cells. S100 expression, bcl2 expression and myeloperoxidase expression in giant-cells epulis are shown in Fig. 2. By immunohistochemistry, 100 % of giant-cells appeared to be positive for myeloperoxidase, whereas only 75,31 % of giant-cells were positive for Bcl 2 (P < 0.05). By the fact s100 protein has expressed in 10,72 % of giant-cells. Myeloperoxidase has physiologically expressed in plasmatic cells of epulis 87,69 %. s100 and bcl 2 have expressed in plasmatic cells 24,34 % (P < 0.05) and 11,28 % respectively, bcl 2 and myeloperoxidase expression being weak or absent in the connective tissue.

The immunoexpression of s100, bcl2 end MPO (Group 2) have been confirmed by the presence of brown stained cytoplasm in cell infiltration. In general, s100 staining was more intensive in the plasmatic cells. In acute inflamation S100 (Fig. 2) only 36,2 ± 5,3 % of the cells appeared to be positive. The cell infiltration, showed bcl2 immunoreactivity figured 76,1 ± 3,3 % (P < 0.05) in acute process (Fig. 2). Myeloperoxidase has expressed in 98,2 ± 5,9 % (P < 0.01) positive cells in acute inflammation.

Figure 2 shows the results of the s100, bcl2 and MPO expression in chronic process.

Myeloperoxidase 82,28 ± 2,5 % P < 0.01 was expressed in chronic plasmatic cell infiltration during inflammation. Bcl 2 was expressed in chronic plasmatic cells infiltration 55,67 ± 6,1 % P < 0.05. Тhe immunoexpression of S100 in cell infiltration has shown the result of 95,0 ± 0.31 % positive cells.

Тhe immunoexpression of s100, bcl2 and MPO in epithelial layers (acute process) have shown the result of 100 %, 82,2 ± 2.93 % and 100 % respectively. In chronic process of inflammation the positive cells have demonstratrd s100 - 100 %, bcl2 – 78,25 ± 4,23 % and myeloperoxidase - 100 % respectively.

## Discussion

This study has claimed that MPO was able to stimulate higher of bcl2 expression in inflammation cells during chronic and acute process. Myeloperoxidase activity expressed in neutrophils recruited to the gingiva after chemical or immunological insults contributes to tissue destruction. Meloperoxidase might influence the extent and/or the severity of periodontal diseases [17].

High level of the MPO have been observed in giant cells. Elevated bcl2 expression levels can prevent cellular apoptosis, thereby inducing inflammatory cells to remain locally in the periodontal tissue, causing consequent excessive cytokine secretion which leads to the progressive destruction of periodontal tissues [1]. Myeloperoxidase can be liberated from activated neutrophils by degranulation only in moderate levels [18], and bcl2 availability protects regions. Cytolysis of neutrophils in the course of inflammation formation could provide a release mechanism [18, 19] and explain the high levels of myeloperoxidase and S100. Activity of S100A12 and C-reactive protein can be markers of inflammatory activity in chronic periodontitis [20]. Previous studies have suggested that apoptosis is involved in the pathogenesis of inflammatory periodontal disease [21]. It has also been demonstrated that the higher frequency of Bcl-2 expression results in progressive periodontal destruction [22].

Gamonall et all. [23] has not found statistical difference in varions amount of bcl2 in healthy gingiva and in gingiva of patients with periodontal disease. Our studies have confirmed the view of Pandilova [24] and Ellis et all. [25] that chronic progression of inflammation decrease expresses bcl-2. In human gingival fibroblasts with inflammation activation of bcl2 has not been observed in different stages of the infection and giant-cell epulis. Sule Bulut et all. [26] results indicate that the pathogenesis of cyclosporin A induced gingival overgrowth might involve inhibition of apoptosis and overexpression of bcl-2 in the setting of high serum cyclosporin A. Our study has demonstrated a high level of bcl2 expression and inhibition of apoptosis in cells of gingival epithelium. We believe this is due to the epithelium protective functions. In gingival epithelium s100 is also related to the differentiation stage [27]. These positive cells are known to activate macrophages or neutrophils [28]. Research by Saito et al. [29] demonstrated numerous bcl-2-positive epithelial cells in gingival biopsies from patients who were taking nifedipine and phenytoin, indicating that this protein might be involved in the development of nifedipine-induced gingival hyperplasia. We believe that the presence of Bcl-2 protein is not an indicator of giant cell epulis favorable courses.

S100 plays a important part in the immune response related to periodontitis. s100 binds 2 Ca2+ and 2 Zn2+ ions. If Zn2+ binds to S100 it decreases its Ca2+ affinity. S100 also interacts with p53 in a Ca2 + −dependent manner, which affects stability of S100-p53 interaction [30] Aforesaid s100-p53 interaction leads to inhibition of apoptosis during inflammation. The Bcl-2 protein is a potent inhibitor of cell death, whereas the wild-type p53 protein activates the apoptotic pathway [5].

Mutated p53 loses this function and allows the proliferation of neoplastic cells. Bcl-2 also modulates the function of p53 and triggers cell proliferation and transformation [31].

Thr expression of S100 have been detected as proinflammatory phagocytes cells at sites of intestinal inflammation [32, 33]. Systemic autoimmune diseases (dermatomyositis, systemic lupus erythematosus, Kawasaki disease etc.) have clear association with S100 expression in macrophages infiltraton with degeneration of tissue [34, 35].

## Conclusion

Investigations on bcl2 marker in gingival cells during periodontal inflammation we suggestion that periodontium tissues, continuously exposed to bacterial infections may contain cells with high level of myeloperoxidase results that damage DNA by product of catalysis.

**Fig 3:**
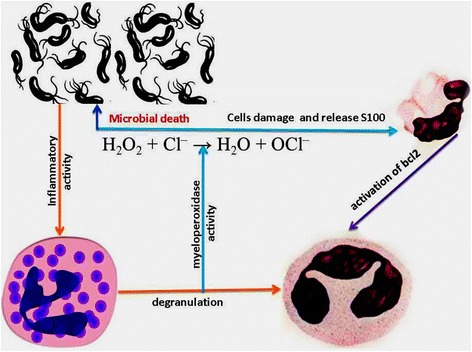
S100, bcl2 and myeloperoxid protein interaction during periodontal inflammation

The highest levels of s100 activity have been found at sites with chronic inflammation. Our results suggest that low s100 expression may play an important role in the activity of giant cells in giant cell epulis. That is considered to be the most significant factors of prognosis in giant cell granuloma. As a result of our research we created a diagram Fig. 3.
